# Supplementary material for: Emojis influence autobiographical memory retrieval from reading words: An fMRI-based study
Source: PLoS One. 2020 Jul 1;15(7):e0234104. doi: 10.1371/journal.pone.0234104 (PMC7329082; doi:10.1371/journal.pone.0234104)
Supplement: S1 Text — Extra file with supplementary information regarding the presented study. (PDF) [file pone.0234104.s001.pdf]

Supplementary material

This is a supplement to the paper *Emojis influence memories from reading words: An fMRI-based study* with the aim of providing extra information and results to the interested reader. References to equations, tables and figures of this part will be given in boldface, to distinguish from references to the main part of the paper.

1 Contrast congruent > incongruent

As mentioned in the main body of the paper the study of the third contrast (c) congruent > incongruent, did not provide any significant activation. No significant activation patterns were detected, except for a small residual within the left ventricles, as it can be observed in Fig.S1. Nor a closer examination over the different subjects at the single-subject level did provide significant results for any of the participants.

Fig. S1: **Significant activation clusters from the group analysis for the considered contrast.** (c) Congruent > Incongruent

2 Blind source separation methods

Conventional analysis of fMRI data is usually conducted within the GLM framework. This assumes that the fMRI measurements of the BOLD signals can be modeled as a convolution between the actual neuronal activation and a particular HRF. Thus, all the GLM-based methods are rooted in the simple assumption of the prior knowledge of the HRF. This assumption is a major limitation of GLM, making it model-dependent and in need of a-priori information by the user. Furthermore, in reality the HRF varies among different subjects as well as among different areas of the same brain (intersubject and intrasubject variability) [1] and the BOLD response also presents non-linear characteristics due to neuronal and vascular changes [2].

Alternatively, blind or semi-blind matrix or tensor factorization methods constitute more attractive alternatives to GLM. Those methods use no or limited prior knowledge, in order to improve the false positive rate in GLM, due to the possibly wrong modelling of the HRF function. Such methods have been also widely used in the analysis of resting state fMRI data, where no prior knowledge of the regressors of interest is available.

Matrix factorization methods are the most well-known alternative to GLM for fMRI data analysis. However, in traditional matrix factorization approaches, such as Independent Component Analysis (ICA), after acquiring a 3D fMRI image, the data are reshaped to a lower dimension, giving rise to a sequence of vectors. These vectors (3D images at different time instants) are stacked together to form a matrix. In this way, the intrinsically 5th-order ($x \times y \times$

$z \times$ time $\times$ subjects) problem of a multi-subject fMRI analysis is transformed into a 2nd-order one. ICA, moreover, relies on the assumption that there exist statistically independent spatial maps (the brain activity patterns), each one corresponding to a time-course, which plays the role of the regressor in the GLM framework. This type of unfolding of higher-order data into two-way arrays leads to decompositions that are non-unique, unless specific assumptions on the involved factors are made. Moreover, it can result in a loss of underlying informative correlations that may exist, because the neighborhood information is not respected. Hence, by definition, such methods fall short in exploiting the inherently multi-way nature of fMRI data.

On the other hand, this is achieved with tensor methods. Tensor models, which are multi-dimensional arrays (generalizations of matrices) have been recently used in task-related fMRI analysis [3, 4] because, among other merits, they i) produce unique representations under mild conditions (modulo scaling and permutation ambiguities), ii) can improve the ability of extracting spatiotemporal modes of interest, and iii) facilitate neurophysiologically meaningful interpretations [3] that may be hidden in the correlations that underlie multi-dimensional data sets. A common assumption in the use of multisubject tensor methods for fMRI analysis, is that the activation patterns of each subject correspond to a similar time-course (regressor). Block Term Decomposition (BTD) [5] approximates the high-order data by a sum of low multilinear rank terms, and provides, with respect to better known Canonical Polyadic Decomposition (CPD) model, higher modeling potential and higher r o spatial overlap among the sources and noise [6].

Hence, in this paper, Group Independent Component Analysis (GICA) [7] and the BTD-based method [5, 4], were employed, in addition to the General Linear Model (GLM). The Infomax [8] ICA algorithm was adopted as implemented in the Group ICA of the fMRI Toolbox (GIFT) [9], a widely used tool for the analysis of fMRI data [7, 10]. The BTD algorithm was implemented in Tensorlab [11].

3 Results of GICA with Infomax

Apart from the standard group analysis using FSL, analysis of the fMRI data using BTD and GICA has been performed. One of the main advantages of the fully blind methods is the fact that an explicit definition of the experimental task-related time-course is not required in order to obtain significant results; these methods learn both the parametric maps and the corresponding activation patters (regressors of interest) from the data themselves.

The blind methods do not require the imposition of any task-related regressor. Thus, we determined the specific sources of interest by selecting the sources whose time-courses present the highest correlation with the studied experimental tasks. Unlike GLM, significant information can be obtained directly using any statistical test, such as t-test or z-scores, without having to define any particular contrast.

Table 1: **GICA results.**

Cluster Size	z score	x	y	z	BA	Anatomical Labels
Words + Emojis						
1464	5.9	-48	24	22	45,46	L M frontal
337	4.0	52	32	14	45,46	R M frontal
	4.0	52	36	10		
Pseudowords						
1884	5.8	32	-68	46	7	R S prefrontal
	3.2	34	-76	18		
	3.2	48	-42	50		
883	4.8	-20	-64	56	7	L S prefrontal
	3.6	-20	-76	44		

Note: Abbreviations as in Table 5.

Clusters with z exceeding 3.0, and associated peak coordinates for the sources that exhibited the highest correlation with the experimental tasks.

Thus, after the selection of the sources of interest, the obtained parameter maps were thresholded for statistical significance at $z > 3.1$ ($p < 0.01$). Fig. 1 shows the significant clusters of GICA (as implemetend in GIFT [9]) for the different considered tasks.

Figure 1: **Significant activation clusters from the analysis using GIFT**. Threshold used for significant activity at $z > 3.1$.

From the results of Infomax, we observe significant activity within the Broca's area in both hemispheres and also some activity within the Inferior parietal cortex. For pseudowords, we observed a significant activation cluster within the superior parietal lobe, which extends along the inferior parietal sulcus, mainly on the right hemisphere.

Concerning pseudowords, apart from a small cluster within the frontal area, the major activation is localized around the superior parietal cortex along the inferior parietal sulcus. Eventually, the corresponding spatial map correlated with all the stimuli resembles the visual cortex, as we expected. Besides, we observe some minor clusters within the prefrontal cortex. It should be also noted that the resulting spatial maps of ICA do not contain dependent (overlapped) areas, e.g., the visual area is not present in the word spatial maps, hence the spatial maps of ICA are not similar with those of FSL.

4 Selection of Words

4.1 Familiarity

Words	Never	(no label)	Rarely	(no label)	Some times	(no label)	Frequent	(no label)	Very frequent	Total	Aver. Rating
Οργή (Rage)	0.00%<br>0	4.65%<br>2	18.60%<br>8	11.63%<br>5	16.28%<br>7	4.65%<br>2	13.95%<br>6	11.63%<br>5	18.60%<br>8	43	5.79
Φίλος (Friend)	2.33%<br>1	2.33%<br>1	0.00%<br>0	0.00%<br>0	6.98%<br>3	2.33%<br>1	23.26%<br>10	18.60%<br>8	44.19%<br>19	43	7.65
Απομόνωση (Isolation)	2.50%<br>1	22.50%<br>9	27.50%<br>11	10.00%<br>4	12.50%<br>5	12.50%<br>5	10.00%<br>4	0.00%<br>0	2.50%<br>1	40	4
Γέννηση (Birth)	0.00%<br>0	16.67%<br>7	21.43%<br>9	11.90%<br>5	21.43%<br>9	14.29%<br>6	7.14%<br>3	4.76%<br>2	2.38%<br>1	42	4.48
Ζωή (Life)	0.00%<br>0	0.00%<br>0	2.38%<br>1	7.14%<br>3	9.52%<br>4	11.90%<br>5	14.29%<br>6	19.05%<br>8	35.71%<br>15	42	7.29
Αντοχή (Resistance)	0.00%<br>0	7.14%<br>3	14.29%<br>6	11.90%<br>5	19.05%<br>8	19.05%<br>8	9.52%<br>4	14.29%<br>6	4.76%<br>2	42	5.38
Εγκατάλειψη (Abandonment)	0.00%<br>0	11.63%<br>5	20.93%<br>9	25.58%<br>11	16.28%<br>7	6.98%<br>3	9.30%<br>4	4.65%<br>2	4.65%<br>2	43	4.56
Αλήθεια (Truth)	0.00%<br>0	2.44%<br>1	4.88%<br>2	4.88%<br>2	12.20%<br>5	17.07%<br>7	26.83%<br>11	19.51%<br>8	12.20%<br>5	41	6.56
Πάθος (Passion)	2.50%<br>1	7.50%<br>3	10.00%<br>4	7.50%<br>3	22.50%<br>9	20.00%<br>8	20.00%<br>8	5.00%<br>2	5.00%<br>2	40	5.35
Χαρά (Joy)	0.00%<br>0	0.00%<br>0	2.44%<br>1	7.32%<br>3	14.63%<br>6	14.63%<br>6	36.59%<br>15	12.20%<br>5	12.20%<br>5	41	6.61
Χωρισμός (Breaking up)	2.44%<br>1	0.00%<br>0	17.07%<br>7	14.63%<br>6	21.95%<br>9	12.20%<br>5	14.63%<br>6	9.76%<br>4	7.32%<br>3	41	5.41
Ειλικρίνεια (Sincerity)	0.00%<br>0	0.00%<br>0	4.88%<br>2	17.07%<br>7	31.71%<br>13	26.83%<br>11	9.76%<br>4	7.32%<br>3	2.44%<br>1	41	5.51
Επιθυμία (Willingness)	0.00%<br>0	0.00%<br>0	7.32%<br>3	9.76%<br>4	14.63%<br>6	9.76%<br>4	26.83%<br>11	24.39%<br>10	7.32%<br>3	41	6.41
Αρρώστια (Illness)	0.00%<br>0	2.33%<br>1	2.33%<br>1	11.63%<br>5	13.95%<br>6	9.30%<br>4	30.23%<br>13	13.95%<br>6	16.28%<br>7	43	6.53
Μελαγχολία (Melancholy)	0.00%<br>0	2.44%<br>1	12.20%<br>5	12.20%<br>5	17.07%<br>7	17.07%<br>7	21.95%<br>9	14.63%<br>6	2.44%<br>1	41	5.71
Βάσανο (Affliction)	2.44%<br>1	14.63%<br>6	24.39%<br>10	9.76%<br>4	9.76%<br>4	12.20%<br>5	4.88%<br>2	9.76%<br>4	12.20%<br>5	41	4.88
Φόνος (Murder)	16.67%<br>7	16.67%<br>7	19.05%<br>8	9.52%<br>4	9.52%<br>4	7.14%<br>3	11.90%<br>5	2.38%<br>1	7.14%<br>3	42	4.02
Απαισιοδοξία (Pessimism)	0.00%<br>0	2.33%<br>1	13.95%<br>6	11.63%<br>5	16.28%<br>7	18.60%<br>8	13.95%<br>6	11.63%<br>5	11.63%<br>5	43	5.81
Τσακωμός (Fight)	0.00%<br>0	10.00%<br>4	7.50%<br>3	2.50%<br>1	10.00%<br>4	7.50%<br>3	27.50%<br>11	25.00%<br>10	10.00%<br>4	40	6.3
Ελπίδα (Hope)	0.00%<br>0	5.00%<br>2	17.50%<br>7	5.00%<br>2	22.50%<br>9	12.50%<br>5	22.50%<br>9	12.50%<br>5	2.50%<br>1	40	5.5
Ψυχή (Soul)	0.00%<br>0	9.52%<br>4	19.05%<br>8	16.67%<br>7	7.14%<br>3	9.52%<br>4	19.05%<br>8	9.52%<br>4	9.52%<br>4	42	5.31
Συντροφιά (Company)	0.00%<br>0	4.76%<br>2	7.14%<br>3	7.14%<br>3	19.05%<br>8	19.05%<br>8	16.67%<br>7	14.29%<br>6	11.90%<br>5	42	6.07

Table 2: Familiarity rating of the words selected

Words	Never	(no label)	Rarely	(no label)	Some times	(no label)	Frequent	(no label)	Very frequent	Total	Aver. Rating
Φιλία (Friendship)	0.00%<br>0	0.00%<br>0	4.76%<br>2	2.38%<br>1	7.14%<br>3	16.67%<br>7	19.05%<br>8	33.33%<br>14	16.67%<br>7	42	7.1
Θάνατος (Death)	2.38%<br>1	4.76%<br>2	7.14%<br>3	14.29%<br>6	9.52%<br>4	14.29%<br>6	28.57%<br>12	16.67%<br>7	2.38%<br>1	42	5.79
Θάρρος (Courage)	0.00%<br>0	4.88%<br>2	9.76%<br>4	19.51%<br>8	31.71%<br>13	7.32%<br>3	17.07%<br>7	7.32%<br>3	2.44%<br>1	41	5.2
Μίσος (Hate)	6.98%<br>3	13.95%<br>6	30.23%<br>13	18.60%<br>8	4.65%<br>2	13.95%<br>6	4.65%<br>2	4.65%<br>2	2.33%<br>1	43	3.98
Ενοχή (Guilt)	0.00%<br>0	20.93%<br>9	11.63%<br>5	13.95%<br>6	16.28%<br>7	23.26%<br>10	11.63%<br>5	2.33%<br>1	0.00%<br>0	43	4.53
Νοσοκομείο (Hospital)	2.33%<br>1	4.65%<br>2	11.63%<br>5	11.63%<br>5	11.63%<br>5	6.98%<br>3	25.58%<br>11	11.63%<br>5	13.95%<br>6	43	5.91
Απόλαυση (Enjoyment)	0.00%<br>0	6.98%<br>3	9.30%<br>4	13.95%<br>6	18.60%<br>8	13.95%<br>6	20.93%<br>9	13.95%<br>6	2.33%<br>1	43	5.53
Ευτυχία (Happiness)	0.00%<br>0	4.76%<br>2	2.38%<br>1	14.29%<br>6	23.81%<br>10	16.67%<br>7	16.67%<br>7	9.52%<br>4	11.90%<br>5	42	5.93
Κλάμα (Cry)	0.00%<br>0	2.44%<br>1	9.76%<br>4	4.88%<br>2	19.51%<br>8	12.20%<br>5	21.95%<br>9	17.07%<br>7	12.20%<br>5	41	6.24
Λύπη (Regret)	0.00%<br>0	0.00%<br>0	0.00%<br>0	14.63%<br>6	12.20%<br>5	12.20%<br>5	36.59%<br>15	14.63%<br>6	9.76%<br>4	41	6.54
Ελευθερία (Freedom)	0.00%<br>0	7.32%<br>3	17.07%<br>7	4.88%<br>2	21.95%<br>9	14.63%<br>6	21.95%<br>9	9.76%<br>4	2.44%<br>1	41	5.37
Θρήνος (Lament)	4.88%<br>2	31.71%<br>13	29.27%<br>12	9.76%<br>4	4.88%<br>2	4.88%<br>2	12.20%<br>5	0.00%<br>0	2.44%<br>1	41	3.56
Γενέθλια (Birthday)	0.00%<br>0	4.76%<br>2	14.29%<br>6	7.14%<br>3	19.05%<br>8	9.52%<br>4	26.19%<br>11	14.29%<br>6	4.76%<br>2	42	5.74
Ανακούφιση (Relief)	0.00%<br>0	9.52%<br>4	11.90%<br>5	11.90%<br>5	28.57%<br>12	23.81%<br>10	9.52%<br>4	2.38%<br>1	2.38%<br>1	42	4.95
Γέλιο (Laugh)	0.00%<br>0	5.00%<br>2	0.00%<br>0	7.50%<br>3	7.50%<br>3	5.00%<br>2	22.50%<br>9	27.50%<br>11	25.00%<br>10	40	7.1
Αγάπη (Love)	0.00%<br>0	0.00%<br>0	2.38%<br>1	2.38%<br>1	7.14%<br>3	4.76%<br>2	21.43%<br>9	26.19%<br>11	35.71%<br>15	42	7.62
Ανθρωπιά (Humanity)	0.00%<br>0	11.90%<br>5	11.90%<br>5	11.90%<br>5	19.05%<br>8	26.19%<br>11	7.14%<br>3	9.52%<br>4	2.38%<br>1	42	5.07
Απιστία (Infidelity)	0.00%<br>0	9.76%<br>4	21.95%<br>9	17.07%<br>7	17.07%<br>7	4.88%<br>2	9.76%<br>4	14.63%<br>6	4.88%<br>2	41	4.98
Αισιοδοξία (Optimism)	0.00%<br>0	4.76%<br>2	11.90%<br>5	7.14%<br>3	33.33%<br>14	14.29%<br>6	19.05%<br>8	9.52%<br>4	0.00%<br>0	42	5.36
Πόνος (Pain)	0.00%<br>0	0.00%<br>0	4.76%<br>2	4.76%<br>2	19.05%<br>8	14.29%<br>6	19.05%<br>8	26.19%<br>11	11.90%<br>5	42	6.64
Σεβασμός (Respect)	0.00%<br>0	4.65%<br>2	4.65%<br>2	6.98%<br>3	11.63%<br>5	20.93%<br>9	23.26%<br>10	13.95%<br>6	13.95%<br>6	43	6.35
Συμπάθεια (Sympathy)	0.00%<br>0	2.44%<br>1	7.32%<br>3	7.32%<br>3	19.51%<br>8	12.20%<br>5	34.15%<br>14	7.32%<br>3	9.76%<br>4	41	6.12
Απελπισία (Desperation)	0.00%<br>0	9.30%<br>4	6.98%<br>3	23.26%<br>10	16.28%<br>7	6.98%<br>3	25.58%<br>11	4.65%<br>2	6.98%<br>3	43	5.35
Απώλεια (Loss)	0.00%<br>0	7.32%<br>3	21.95%<br>9	17.07%<br>7	21.95%<br>9	7.32%<br>3	17.07%<br>7	7.32%<br>3	0.00%<br>0	41	4.8
Πένθος (Mourning)	4.76%<br>2	7.14%<br>3	23.81%<br>10	21.43%<br>9	16.67%<br>7	9.52%<br>4	11.90%<br>5	2.38%<br>1	2.38%<br>1	42	4.4
Φόβος (Fear)	0.00%<br>0	0.00%<br>0	7.14%<br>3	4.76%<br>2	14.29%<br>6	16.67%<br>7	26.19%<br>11	21.43%<br>9	9.52%<br>4	42	6.52
Έρωτας (Love)	0.00%<br>0	0.00%<br>0	6.82%<br>3	2.27%<br>1	6.82%<br>3	15.91%<br>7	31.82%<br>14	15.91%<br>7	20.45%<br>9	44	6.93

Table 3: Familiarity rating of the words selected

4.2 Imageability

Words	Very difficult	(no label)	Difficult	(no label)	Neutral	(no label)	Easy	(no label)	Very easy	Total	Aver. Rating
Οργή (Rage)	0.00%<br>0	2.50%<br>1	7.50%<br>3	5.00%<br>2	22.50%<br>9	12.50%<br>5	37.50%<br>15	7.50%<br>3	5.00%<br>2	40	6.03
Φίλος (Friend)	0.00%<br>0	2.50%<br>1	0.00%<br>0	2.50%<br>1	5.00%<br>2	2.50%<br>1	22.50%<br>9	15.00%<br>6	50.00%<br>20	40	7.83
Απομόνωση (Isolation)	7.32%<br>3	4.88%<br>2	34.15%<br>14	14.63%<br>6	12.20%<br>5	12.20%<br>5	9.76%<br>4	2.44%<br>1	2.44%<br>1	41	4.22
Γέννηση (Birth)	2.50%<br>1	0.00%<br>0	7.50%<br>3	10.00%<br>4	15.00%<br>6	15.00%<br>6	30.00%<br>12	10.00%<br>4	10.00%<br>4	40	6.1
Ζωή (Life)	0.00%<br>0	0.00%<br>0	2.56%<br>1	2.56%<br>1	5.13%<br>2	7.69%<br>3	15.38%<br>6	7.69%<br>3	58.97%<br>23	39	7.9
Αντοχή (Resistance)	0.00%<br>0	10.53%<br>4	10.53%<br>4	10.53%<br>4	15.79%<br>6	7.89%<br>3	10.53%<br>4	13.16%<br>5	21.05%<br>8	38	5.89
Εγκατάλειψη (Abandonment)	0.00%<br>0	0.00%<br>0	0.00%<br>0	5.26%<br>2	7.89%<br>3	13.16%<br>5	28.95%<br>11	23.68%<br>9	21.05%<br>8	38	7.21
Αλήθεια (Truth)	5.26%<br>2	10.53%<br>4	13.16%<br>5	15.79%<br>6	28.95%<br>11	7.89%<br>3	2.63%<br>1	13.16%<br>5	2.63%<br>1	38	4.68
Πάθος (Passion)	2.70%<br>1	0.00%<br>0	18.92%<br>7	13.51%<br>5	16.22%<br>6	13.51%<br>5	18.92%<br>7	13.51%<br>5	2.70%<br>1	37	5.41
Χαρά (Joy)	10.53%<br>4	23.68%<br>9	23.68%<br>9	2.63%<br>1	21.05%<br>8	7.89%<br>3	5.26%<br>2	0.00%<br>0	5.26%<br>2	38	3.76
Χωρισμός (Breaking up)	0.00%<br>0	7.89%<br>3	5.26%<br>2	2.63%<br>1	5.26%<br>2	10.53%<br>4	21.05%<br>8	23.68%<br>9	23.68%<br>9	38	6.82
Ειλικρίνεια (Sincerity)	0.00%<br>0	0.00%<br>0	0.00%<br>0	0.00%<br>0	0.00%<br>0	5.26%<br>2	23.68%<br>9	34.21%<br>13	36.84%<br>14	38	8.03
Επιθυμία (Willingness)	5.26%<br>2	5.26%<br>2	0.00%<br>0	2.63%<br>1	15.79%<br>6	13.16%<br>5	23.68%<br>9	13.16%<br>5	21.05%<br>8	38	6.45
Αρρώστια (Illness)	10.00%<br>4	22.50%<br>9	15.00%<br>6	10.00%<br>4	15.00%<br>6	10.00%<br>4	10.00%<br>4	2.50%<br>1	5.00%<br>2	40	4.1
Μελαγχολία (Melancholy)	7.89%<br>3	15.79%<br>6	13.16%<br>5	10.53%<br>4	23.68%<br>9	7.89%<br>3	7.89%<br>3	7.89%<br>3	5.26%<br>2	38	4.53
Βάσανο (Affliction)	0.00%<br>0	0.00%<br>0	10.53%<br>4	2.63%<br>1	13.16%<br>5	18.42%<br>7	26.32%<br>10	13.16%<br>5	15.79%<br>6	38	6.5
Φόνος (Murder)	2.63%<br>1	15.79%<br>6	21.05%<br>8	7.89%<br>3	21.05%<br>8	7.89%<br>3	13.16%<br>5	5.26%<br>2	5.26%<br>2	38	4.63
Απαισιοδοξία (Pessimism)	2.63%<br>1	2.63%<br>1	2.63%<br>1	7.89%<br>3	7.89%<br>3	15.79%<br>6	18.42%<br>7	23.68%<br>9	18.42%<br>7	38	6.66
Τσακωμός (Fight)	2.63%<br>1	13.16%<br>5	18.42%<br>7	10.53%<br>4	31.58%<br>12	7.89%<br>3	5.26%<br>2	7.89%<br>3	2.63%<br>1	38	4.55
Ελπίδα (Hope)	0.00%<br>0	0.00%<br>0	0.00%<br>0	0.00%<br>0	0.00%<br>0	13.16%<br>5	23.68%<br>9	31.58%<br>12	31.58%<br>12	38	7.82
Ψυχή (Soul)	7.89%<br>3	10.53%<br>4	7.89%<br>3	10.53%<br>4	26.32%<br>10	5.26%<br>2	15.79%<br>6	10.53%<br>4	5.26%<br>2	38	5
Συντροφιά (Company)	25.64%<br>10	17.95%<br>7	17.95%<br>7	12.82%<br>5	10.26%<br>4	0.00%<br>0	5.13%<br>2	2.56%<br>1	7.69%<br>3	39	3.44

Table 4: Imageability rating of the words selected

Words	Very difficult	(no label)	Difficult	(no label)	Neutral	(no label)	Easy	(no label)	Very easy	Total	Aver. Rating
Φιλία (Friendship)	0.00%<br>0	0.00%<br>0	0.00%<br>0	2.63%<br>1	2.63%<br>1	18.42%<br>7	18.42%<br>7	23.68%<br>9	34.21%<br>13	38	7.61
Θάνατος (Death)	0.00%<br>0	2.63%<br>1	5.26%<br>2	5.26%<br>2	2.63%<br>1	2.63%<br>1	18.42%<br>7	34.21%<br>13	28.95%<br>11	38	7.34
Θάρρος (Courage)	0.00%<br>0	0.00%<br>0	2.63%<br>1	7.89%<br>3	5.26%<br>2	2.63%<br>1	21.05%<br>8	28.95%<br>11	31.58%<br>12	38	7.45
Μίσος (Hate)	0.00%<br>0	8.11%<br>3	10.81%<br>4	21.62%<br>8	13.51%<br>5	10.81%<br>4	21.62%<br>8	10.81%<br>4	2.70%<br>1	37	5.3
Ενοχή (Guilt)	2.63%<br>1	13.16%<br>5	26.32%<br>10	7.89%<br>3	18.42%<br>7	10.53%<br>4	13.16%<br>5	2.63%<br>1	5.26%<br>2	38	4.55
Νοσοκομείο (Hospital)	0.00%<br>0	0.00%<br>0	0.00%<br>0	0.00%<br>0	2.63%<br>1	0.00%<br>0	21.05%<br>8	10.53%<br>4	65.79%<br>25	38	8.37
Απόλαυση (Enjoyment)	2.63%<br>1	5.26%<br>2	0.00%<br>0	5.26%<br>2	10.53%<br>4	13.16%<br>5	18.42%<br>7	28.95%<br>11	15.79%<br>6	38	6.68
Ευτυχία (Happiness)	0.00%<br>0	0.00%<br>0	2.63%<br>1	5.26%<br>2	21.05%<br>8	5.26%<br>2	23.68%<br>9	21.05%<br>8	21.05%<br>8	38	6.89
Κλάμα (Cry)	0.00%<br>0	0.00%<br>0	0.00%<br>0	0.00%<br>0	0.00%<br>0	0.00%<br>0	10.53%<br>4	23.68%<br>9	65.79%<br>25	38	8.55
Λύπη (Regret)	0.00%<br>0	2.63%<br>1	2.63%<br>1	5.26%<br>2	2.63%<br>1	7.89%<br>3	34.21%<br>13	21.05%<br>8	23.68%<br>9	38	7.16
Ελευθερία (Freedom)	10.53%<br>4	10.53%<br>4	7.89%<br>3	7.89%<br>3	26.32%<br>10	10.53%<br>4	10.53%<br>4	7.89%<br>3	7.89%<br>3	38	4.89
Θρήνος (Lament)	0.00%<br>0	0.00%<br>0	7.89%<br>3	7.89%<br>3	18.42%<br>7	5.26%<br>2	23.68%<br>9	15.79%<br>6	21.05%<br>8	38	6.61
Γενέθλια (Birthday)	0.00%<br>0	0.00%<br>0	0.00%<br>0	0.00%<br>0	5.26%<br>2	2.63%<br>1	7.89%<br>3	7.89%<br>3	76.32%<br>29	38	8.47
Ανακούφιση (Relief)	2.56%<br>1	5.13%<br>2	5.13%<br>2	12.82%<br>5	23.08%<br>9	7.69%<br>3	15.38%<br>6	15.38%<br>6	12.82%<br>5	39	5.87
Γέλιο (Laugh)	0.00%<br>0	0.00%<br>0	0.00%<br>0	0.00%<br>0	0.00%<br>0	2.63%<br>1	7.89%<br>3	7.89%<br>3	81.58%<br>31	38	8.68
Αγάπη (Love)	0.00%<br>0	5.26%<br>2	0.00%<br>0	2.63%<br>1	7.89%<br>3	2.63%<br>1	15.79%<br>6	13.16%<br>5	52.63%<br>20	38	7.66
Ανθρωπιά (Humanity)	5.26%<br>2	7.89%<br>3	10.53%<br>4	5.26%<br>2	10.53%<br>4	13.16%<br>5	10.53%<br>4	15.79%<br>6	21.05%<br>8	38	5.95
Απιστία (Infidelity)	5.13%<br>2	0.00%<br>0	12.82%<br>5	10.26%<br>4	7.69%<br>3	15.38%<br>6	28.21%<br>11	12.82%<br>5	7.69%<br>3	39	5.85
Αισιοδοξία (Optimism)	5.13%<br>2	5.13%<br>2	17.95%<br>7	10.26%<br>4	15.38%<br>6	12.82%<br>5	15.38%<br>6	12.82%<br>5	5.13%<br>2	39	5.21
Πόνος (Pain)	0.00%<br>0	0.00%<br>0	2.63%<br>1	0.00%<br>0	15.79%<br>6	18.42%<br>7	26.32%<br>10	23.68%<br>9	13.16%<br>5	38	6.89
Σεβασμός (Respect)	5.26%<br>2	21.05%<br>8	21.05%<br>8	13.16%<br>5	13.16%<br>5	5.26%<br>2	10.53%<br>4	7.89%<br>3	2.63%<br>1	38	4.21
Συμπάθεια (Sympathy)	5.26%<br>2	18.42%<br>7	13.16%<br>5	10.53%<br>4	10.53%<br>4	10.53%<br>4	21.05%<br>8	7.89%<br>3	2.63%<br>1	38	4.74
Απελπισία (Desperation)	2.44%<br>1	21.95%<br>9	4.88%<br>2	7.32%<br>3	26.83%<br>11	9.76%<br>4	14.63%<br>6	7.32%<br>3	4.88%<br>2	41	4.88
Απώλεια (Loss)	0.00%<br>0	13.16%<br>5	10.53%<br>4	13.16%<br>5	15.79%<br>6	7.89%<br>3	13.16%<br>5	13.16%<br>5	13.16%<br>5	38	5.53
Πένθος (Mourning)	0.00%<br>0	2.56%<br>1	2.56%<br>1	5.13%<br>2	15.38%<br>6	10.26%<br>4	23.08%<br>9	17.95%<br>7	23.08%<br>9	39	6.85
Φόβος (Fear)	2.56%<br>1	2.56%<br>1	5.13%<br>2	5.13%<br>2	10.26%<br>4	5.13%<br>2	20.51%<br>8	28.21%<br>11	20.51%<br>8	39	6.79
Έρωτας (Love)	0.00%<br>0	0.00%<br>0	2.50%<br>1	0.00%<br>0	7.50%<br>3	5.00%<br>2	15.00%<br>6	20.00%<br>8	50.00%<br>20	40	7.9

Table 5: Imageability rating of the words selected

4.3 Arousal

Words	Very big arousal	(no label)	Arousal	(no label)	Neutral	(no label)	Calm	(no label)	Really calm	Total	Aver. Rating
Οργή (Rage)	40.00%<br>14	34.29%<br>12	17.14%<br>6	0.00%<br>0	8.57%<br>3	0.00%<br>0	0.00%<br>0	0.00%<br>0	0.00%<br>0	35	2.03
Φίλος (Friend)	45.71%<br>16	28.57%<br>10	20.00%<br>7	5.71%<br>2	0.00%<br>0	0.00%<br>0	0.00%<br>0	0.00%<br>0	0.00%<br>0	35	1.86
Απομόνωση (Isolation)	8.82%<br>3	5.88%<br>2	17.65%<br>6	8.82%<br>3	14.71%<br>5	0.00%<br>0	26.47%<br>9	5.88%<br>2	11.76%<br>4	34	5.21
Γέννηση (Birth)	22.86%<br>8	20.00%<br>7	25.71%<br>9	8.57%<br>3	14.29%<br>5	2.86%<br>1	2.86%<br>1	0.00%<br>0	2.86%<br>1	35	3.09
Ζωή (Life)	2.94%<br>1	8.82%<br>3	23.53%<br>8	11.76%<br>4	14.71%<br>5	17.65%<br>6	2.94%<br>1	5.88%<br>2	11.76%<br>4	34	4.91
Αντοχή (Resistance)	16.67%<br>6	19.44%<br>7	22.22%<br>8	11.11%<br>4	16.67%<br>6	2.78%<br>1	2.78%<br>1	2.78%<br>1	5.56%<br>2	36	3.58
Εγκατάλειψη (Abandonment)	31.43%<br>11	17.14%<br>6	5.71%<br>2	0.00%<br>0	14.29%<br>5	5.71%<br>2	11.43%<br>4	5.71%<br>2	8.57%<br>3	35	3.91
Αλήθεια (Truth)	25.71%<br>9	17.14%<br>6	31.43%<br>11	5.71%<br>2	8.57%<br>3	2.86%<br>1	0.00%<br>0	2.86%<br>1	5.71%<br>2	35	3.11
Πάθος (Passion)	38.89%<br>14	33.33%<br>12	13.89%<br>5	5.56%<br>2	2.78%<br>1	2.78%<br>1	0.00%<br>0	0.00%<br>0	2.78%<br>1	36	2.25
Χαρά (Joy)	11.11%<br>4	11.11%<br>4	22.22%<br>8	2.78%<br>1	36.11%<br>13	5.56%<br>2	5.56%<br>2	5.56%<br>2	0.00%<br>0	36	4.08
Χωρισμός (Breaking up)	30.56%<br>11	13.89%<br>5	22.22%<br>8	11.11%<br>4	8.33%<br>3	2.78%<br>1	0.00%<br>0	5.56%<br>2	5.56%<br>2	36	3.22
Ειλικρίνεια (Sincerity)	8.82%<br>3	2.94%<br>1	17.65%<br>6	0.00%<br>0	14.71%<br>5	11.76%<br>4	23.53%<br>8	8.82%<br>3	11.76%<br>4	34	5.53
Επιθυμία (Willingness)	33.33%<br>12	11.11%<br>4	22.22%<br>8	8.33%<br>3	0.00%<br>0	5.56%<br>2	5.56%<br>2	5.56%<br>2	8.33%<br>3	36	3.47
Αρρώστια (Illness)	31.43%<br>11	25.71%<br>9	25.71%<br>9	8.57%<br>3	0.00%<br>0	0.00%<br>0	0.00%<br>0	0.00%<br>0	8.57%<br>3	35	2.71
Μελαγχολία (Melancholy)	0.00%<br>0	5.88%<br>2	20.59%<br>7	8.82%<br>3	23.53%<br>8	5.88%<br>2	11.76%<br>4	8.82%<br>3	14.71%<br>5	34	5.47
Βάσανο (Affliction)	8.57%<br>3	8.57%<br>3	48.57%<br>17	11.43%<br>4	8.57%<br>3	2.86%<br>1	5.71%<br>2	2.86%<br>1	2.86%<br>1	35	3.66
Φόνος (Murder)	8.57%<br>3	25.71%<br>9	28.57%<br>10	8.57%<br>3	14.29%<br>5	11.43%<br>4	0.00%<br>0	2.86%<br>1	0.00%<br>0	35	3.43
Απαισιοδοξία (Pessimism)	2.86%<br>1	0.00%<br>0	20.00%<br>7	14.29%<br>5	17.14%<br>6	8.57%<br>3	22.86%<br>8	5.71%<br>2	8.57%<br>3	35	5.4
Τσακωμός (Fight)	51.43%<br>18	20.00%<br>7	20.00%<br>7	2.86%<br>1	0.00%<br>0	0.00%<br>0	0.00%<br>0	2.86%<br>1	2.86%<br>1	35	2.11
Ελπίδα (Hope)	0.00%<br>0	8.57%<br>3	28.57%<br>10	11.43%<br>4	14.29%<br>5	8.57%<br>3	11.43%<br>4	5.71%<br>2	11.43%<br>4	35	5
Ψυχή (Soul)	14.29%<br>5	11.43%<br>4	14.29%<br>5	11.43%<br>4	14.29%<br>5	11.43%<br>4	8.57%<br>3	5.71%<br>2	8.57%<br>3	35	4.49
Συντροφιά (Company)	5.71%<br>2	0.00%<br>0	5.71%<br>2	8.57%<br>3	48.57%<br>17	0.00%<br>0	20.00%<br>7	0.00%<br>0	11.43%<br>4	35	5.43

Table 6: Arousal rating of the words selected

Words	Very difficult	(no label)	Difficult	(no label)	Neutral	(no label)	Easy	(no label)	Very easy	Total	Aver. Rating
Φιλία (Friendship)	14.29%<br>5	5.71%<br>2	14.29%<br>5	0.00%<br>0	8.57%<br>3	8.57%<br>3	20.00%<br>7	17.14%<br>6	11.43%<br>4	35	5.43
Θάνατος (Death)	17.65%<br>6	8.82%<br>3	11.76%<br>4	0.00%<br>0	8.82%<br>3	5.88%<br>2	29.41%<br>10	5.88%<br>2	11.76%<br>4	34	5.09
Θάρρος (Courage)	48.57%<br>17	22.86%<br>8	11.43%<br>4	0.00%<br>0	5.71%<br>2	0.00%<br>0	2.86%<br>1	2.86%<br>1	5.71%<br>2	35	2.51
Μίσος (Hate)	17.14%<br>6	14.29%<br>5	42.86%<br>15	2.86%<br>1	17.14%<br>6	2.86%<br>1	2.86%<br>1	0.00%<br>0	0.00%<br>0	35	3.09
Ενοχή (Guilt)	38.89%<br>14	22.22%<br>8	16.67%<br>6	2.78%<br>1	5.56%<br>2	5.56%<br>2	2.78%<br>1	2.78%<br>1	2.78%<br>1	36	2.72
Νοσοκομείο (Hospital)	25.71%<br>9	17.14%<br>6	25.71%<br>9	8.57%<br>3	14.29%<br>5	2.86%<br>1	0.00%<br>0	0.00%<br>0	5.71%<br>2	35	3.11
Απόλαυση (Enjoyment)	20.00%<br>7	8.57%<br>3	28.57%<br>10	8.57%<br>3	2.86%<br>1	8.57%<br>3	11.43%<br>4	5.71%<br>2	5.71%<br>2	35	4
Ευτυχία (Happiness)	37.14%<br>13	14.29%<br>5	11.43%<br>4	5.71%<br>2	0.00%<br>0	2.86%<br>1	8.57%<br>3	5.71%<br>2	14.29%<br>5	35	3.74
Κλάμα (Cry)	8.33%<br>3	27.78%<br>10	41.67%<br>15	11.11%<br>4	2.78%<br>1	0.00%<br>0	0.00%<br>0	0.00%<br>0	8.33%<br>3	36	3.22
Λύπη (Regret)	17.65%<br>6	5.88%<br>2	17.65%<br>6	2.94%<br>1	17.65%<br>6	5.88%<br>2	5.88%<br>2	8.82%<br>3	17.65%<br>6	34	4.88
Ελευθερία (Freedom)	37.14%<br>13	14.29%<br>5	34.29%<br>12	2.86%<br>1	0.00%<br>0	0.00%<br>0	0.00%<br>0	0.00%<br>0	11.43%<br>4	35	2.83
Θρήνος (Lament)	14.71%<br>5	8.82%<br>3	52.94%<br>18	5.88%<br>2	5.88%<br>2	0.00%<br>0	2.94%<br>1	2.94%<br>1	5.88%<br>2	34	3.41
Γενέθλια (Birthday)	2.78%<br>1	11.11%<br>4	33.33%<br>12	11.11%<br>4	36.11%<br>13	0.00%<br>0	2.78%<br>1	2.78%<br>1	0.00%<br>0	36	3.92
Ανακούφιση (Relief)	8.57%<br>3	8.57%<br>3	5.71%<br>2	14.29%<br>5	2.86%<br>1	5.71%<br>2	14.29%<br>5	14.29%<br>5	25.71%<br>9	35	5.94
Γέλιο (Laugh)	31.43%<br>11	14.29%<br>5	20.00%<br>7	11.43%<br>4	2.86%<br>1	2.86%<br>1	8.57%<br>3	5.71%<br>2	2.86%<br>1	35	3.29
Αγάπη (Love)	25.00%<br>9	16.67%<br>6	11.11%<br>4	5.56%<br>2	5.56%<br>2	2.78%<br>1	8.33%<br>3	2.78%<br>1	22.22%<br>8	36	4.39
Ανθρωπιά (Humanity)	11.43%<br>4	2.86%<br>1	14.29%<br>5	2.86%<br>1	20.00%<br>7	8.57%<br>3	22.86%<br>8	8.57%<br>3	8.57%<br>3	35	5.29
Απιστία (Infidelity)	31.43%<br>11	17.14%<br>6	20.00%<br>7	11.43%<br>4	11.43%<br>4	0.00%<br>0	0.00%<br>0	2.86%<br>1	5.71%<br>2	35	3.03
Αισιοδοξία (Optimism)	20.00%<br>7	5.71%<br>2	20.00%<br>7	5.71%<br>2	14.29%<br>5	2.86%<br>1	14.29%<br>5	11.43%<br>4	5.71%<br>2	35	4.46
Πόνος (Pain)	30.56%<br>11	25.00%<br>9	27.78%<br>10	5.56%<br>2	5.56%<br>2	5.56%<br>2	0.00%<br>0	0.00%<br>0	0.00%<br>0	36	2.47
Σεβασμός (Respect)	2.86%<br>1	0.00%<br>0	14.29%<br>5	5.71%<br>2	28.57%<br>10	5.71%<br>2	22.86%<br>8	8.57%<br>3	11.43%<br>4	35	5.77
Συμπάθεια (Sympathy)	0.00%<br>0	8.57%<br>3	17.14%<br>6	5.71%<br>2	25.71%<br>9	11.43%<br>4	14.29%<br>5	8.57%<br>3	8.57%<br>3	35	5.34
Απελπισία (Desperation)	17.65%<br>6	14.71%<br>5	26.47%<br>9	17.65%<br>6	11.76%<br>4	0.00%<br>0	2.94%<br>1	5.88%<br>2	2.94%<br>1	34	3.5
Απώλεια (Loss)	36.11%<br>13	25.00%<br>9	13.89%<br>5	5.56%<br>2	8.33%<br>3	0.00%<br>0	2.78%<br>1	0.00%<br>0	8.33%<br>3	36	2.86
Πένθος (Mourning)	25.71%<br>9	17.14%<br>6	22.86%<br>8	8.57%<br>3	0.00%<br>0	0.00%<br>0	8.57%<br>3	5.71%<br>2	11.43%<br>4	35	3.71
Φόβος (Fear)	20.00%<br>7	20.00%<br>7	40.00%<br>14	11.43%<br>4	0.00%<br>0	0.00%<br>0	2.86%<br>1	0.00%<br>0	5.71%<br>2	35	2.97
Έρωτας (Love)	50.00%<br>18	16.67%<br>6	19.44%<br>7	2.78%<br>1	0.00%<br>0	2.78%<br>1	0.00%<br>0	2.78%<br>1	5.56%<br>2	36	2.4166

Table 7: Arousal rating of the words selected

4.4 Valence

Words	Very Positive	(no label)	Positive	(no label)	Neutral	(no label)	Negative	(no label)	Very Negative	Total	Aver. Rating
Οργή (Rage)	55.56%<br>20	11.11%<br>4	19.44%<br>7	2.78%<br>1	5.56%<br>2	0.00%<br>0	2.78%<br>1	0.00%<br>0	2.78%<br>1	36	2.19
Φίλος (Friend)	14.29%<br>5	11.43%<br>4	14.29%<br>5	5.71%<br>2	48.57%<br>17	2.86%<br>1	2.86%<br>1	0.00%<br>0	0.00%<br>0	35	3.83
Απομόνωση (Isolation)	5.71%<br>2	0.00%<br>0	2.86%<br>1	0.00%<br>0	0.00%<br>0	2.86%<br>1	5.71%<br>2	20.00%<br>7	62.86%<br>22	35	7.97
Γέννηση (Birth)	31.43%<br>11	22.86%<br>8	17.14%<br>6	14.29%<br>5	2.86%<br>1	5.71%<br>2	0.00%<br>0	0.00%<br>0	5.71%<br>2	35	2.86
Ζωή (Life)	2.86%<br>1	0.00%<br>0	0.00%<br>0	0.00%<br>0	0.00%<br>0	11.43%<br>4	22.86%<br>8	37.14%<br>13	25.71%<br>9	35	7.6
Αντοχή (Resistance)	42.86%<br>15	31.43%<br>11	17.14%<br>6	2.86%<br>1	2.86%<br>1	0.00%<br>0	2.86%<br>1	0.00%<br>0	0.00%<br>0	35	2.03
Εγκατάλειψη (Abandonment)	2.86%<br>1	0.00%<br>0	0.00%<br>0	0.00%<br>0	2.86%<br>1	5.71%<br>2	40.00%<br>14	31.43%<br>11	17.14%<br>6	35	7.37
Αλήθεια (Truth)	5.71%<br>2	0.00%<br>0	0.00%<br>0	0.00%<br>0	11.43%<br>4	2.86%<br>1	31.43%<br>11	34.29%<br>12	14.29%<br>5	35	7.03
Πάθος (Passion)	42.86%<br>15	17.14%<br>6	17.14%<br>6	11.43%<br>4	5.71%<br>2	2.86%<br>1	0.00%<br>0	0.00%<br>0	2.86%<br>1	35	2.46
Χαρά (Joy)	5.71%<br>2	2.86%<br>1	0.00%<br>0	0.00%<br>0	0.00%<br>0	0.00%<br>0	20.00%<br>7	22.86%<br>8	48.57%<br>17	35	7.71
Χωρισμός (Breaking up)	2.86%<br>1	0.00%<br>0	0.00%<br>0	0.00%<br>0	5.71%<br>2	0.00%<br>0	34.29%<br>12	40.00%<br>14	17.14%<br>6	35	7.46
Ειλικρίνεια (Sincerity)	17.14%<br>6	11.43%<br>4	28.57%<br>10	14.29%<br>5	20.00%<br>7	5.71%<br>2	2.86%<br>1	0.00%<br>0	0.00%<br>0	35	3.37
Επιθυμία (Willingness)	0.00%<br>0	0.00%<br>0	2.94%<br>1	0.00%<br>0	0.00%<br>0	0.00%<br>0	14.71%<br>5	23.53%<br>8	58.82%<br>20	34	8.29
Αρρώστια (Illness)	41.67%<br>15	22.22%<br>8	13.89%<br>5	13.89%<br>5	0.00%<br>0	5.56%<br>2	0.00%<br>0	2.78%<br>1	0.00%<br>0	36	2.39
Μελαγχολία (Melancholy)	57.14%<br>20	25.71%<br>9	11.43%<br>4	2.86%<br>1	0.00%<br>0	0.00%<br>0	0.00%<br>0	0.00%<br>0	2.86%<br>1	35	1.8
Βάσανο (Affliction)	2.86%<br>1	2.86%<br>1	0.00%<br>0	0.00%<br>0	5.71%<br>2	5.71%<br>2	2.86%<br>1	34.29%<br>12	45.71%<br>16	35	7.77
Φόνος (Murder)	38.89%<br>14	22.22%<br>8	25.00%<br>9	2.78%<br>1	2.78%<br>1	2.78%<br>1	2.78%<br>1	2.78%<br>1	0.00%<br>0	36	2.42
Απαισιοδοξία (Pessimism)	2.78%<br>1	0.00%<br>0	0.00%<br>0	2.78%<br>1	11.11%<br>4	0.00%<br>0	44.44%<br>16	30.56%<br>11	8.33%<br>3	36	7
Τσακωμός (Fight)	11.43%<br>4	20.00%<br>7	45.71%<br>16	8.57%<br>3	5.71%<br>2	5.71%<br>2	2.86%<br>1	0.00%<br>0	0.00%<br>0	35	3.06
Ελπίδα (Hope)	2.94%<br>1	0.00%<br>0	0.00%<br>0	0.00%<br>0	0.00%<br>0	0.00%<br>0	11.76%<br>4	5.88%<br>2	79.41%<br>27	34	8.47
Ψυχή (Soul)	0.00%<br>0	0.00%<br>0	0.00%<br>0	0.00%<br>0	2.86%<br>1	5.71%<br>2	40.00%<br>14	37.14%<br>13	14.29%<br>5	35	7.54
Συντροφιά (Company)	44.12%<br>15	11.76%<br>4	41.18%<br>14	0.00%<br>0	0.00%<br>0	0.00%<br>0	0.00%<br>0	0.00%<br>0	2.94%<br>1	34	2.18

Table 8: Valence rating of the words selected

Words	Very Positive	(no label)	Positive	(no label)	Neutral	(no label)	Negative	(no label)	Very Negative	Total	Aver. Rating
Φιλία (Friendship)	71.43%<br>25	8.57%<br>3	11.43%<br>4	0.00%<br>0	0.00%<br>0	2.86%<br>1	0.00%<br>0	2.86%<br>1	2.86%<br>1	35	1.89
Θάνατος (Death)	40.00%<br>14	28.57%<br>10	25.71%<br>9	0.00%<br>0	2.86%<br>1	0.00%<br>0	2.86%<br>1	0.00%<br>0	0.00%<br>0	35	2.09
Θάρρος (Courage)	5.71%<br>2	0.00%<br>0	0.00%<br>0	0.00%<br>0	0.00%<br>0	0.00%<br>0	5.71%<br>2	5.71%<br>2	82.86%<br>29	35	8.37
Μίσος (Hate)	65.71%<br>23	20.00%<br>7	5.71%<br>2	0.00%<br>0	0.00%<br>0	0.00%<br>0	0.00%<br>0	0.00%<br>0	8.57%<br>3	35	2
Ενοχή (Guilt)	13.89%<br>5	25.00%<br>9	27.78%<br>10	11.11%<br>4	16.67%<br>6	0.00%<br>0	2.78%<br>1	0.00%<br>0	2.78%<br>1	36	3.19
Νοσοκομείο (Hospital)	6.06%<br>2	0.00%<br>0	0.00%<br>0	0.00%<br>0	3.03%<br>1	0.00%<br>0	15.15%<br>5	27.27%<br>9	48.48%<br>16	33	7.82
Απόλαυση (Enjoyment)	2.86%<br>1	2.86%<br>1	0.00%<br>0	0.00%<br>0	14.29%<br>5	5.71%<br>2	20.00%<br>7	17.14%<br>6	37.14%<br>13	35	7.26
Ευτυχία (Happiness)	42.86%<br>15	22.86%<br>8	25.71%<br>9	5.71%<br>2	0.00%<br>0	0.00%<br>0	0.00%<br>0	0.00%<br>0	2.86%<br>1	35	2.14
Κλάμα (Cry)	0.00%<br>0	0.00%<br>0	0.00%<br>0	0.00%<br>0	20.00%<br>7	14.29%<br>5	42.86%<br>15	14.29%<br>5	8.57%<br>3	35	6.77
Λύπη (Regret)	41.67%<br>15	22.22%<br>8	22.22%<br>8	0.00%<br>0	11.11%<br>4	0.00%<br>0	0.00%<br>0	0.00%<br>0	2.78%<br>1	36	2.33
Ελευθερία (Freedom)	0.00%<br>0	0.00%<br>0	0.00%<br>0	2.86%<br>1	2.86%<br>1	8.57%<br>3	42.86%<br>15	25.71%<br>9	17.14%<br>6	35	7.37
Θρήνος (Lament)	11.43%<br>4	14.29%<br>5	37.14%<br>13	17.14%<br>6	20.00%<br>7	0.00%<br>0	0.00%<br>0	0.00%<br>0	0.00%<br>0	35	3.2
Γενέθλια (Birthday)	34.29%<br>12	28.57%<br>10	25.71%<br>9	2.86%<br>1	2.86%<br>1	2.86%<br>1	0.00%<br>0	0.00%<br>0	2.86%<br>1	35	2.37
Ανακούφιση (Relief)	48.57%<br>17	34.29%<br>12	14.29%<br>5	0.00%<br>0	0.00%<br>0	0.00%<br>0	0.00%<br>0	0.00%<br>0	2.86%<br>1	35	1.86
Γέλιο (Laugh)	82.86%<br>29	8.57%<br>3	2.86%<br>1	0.00%<br>0	2.86%<br>1	0.00%<br>0	0.00%<br>0	0.00%<br>0	2.86%<br>1	35	1.49
Αγάπη (Love)	42.86%<br>15	31.43%<br>11	14.29%<br>5	2.86%<br>1	0.00%<br>0	0.00%<br>0	2.86%<br>1	2.86%<br>1	2.86%<br>1	35	2.29
Ανθρωπιά (Humanity)	0.00%<br>0	0.00%<br>0	0.00%<br>0	0.00%<br>0	2.86%<br>1	17.14%<br>6	40.00%<br>14	28.57%<br>10	11.43%<br>4	35	7.29
Απιστία (Infidelity)	42.86%<br>15	14.29%<br>5	25.71%<br>9	11.43%<br>4	0.00%<br>0	0.00%<br>0	2.86%<br>1	0.00%<br>0	2.86%<br>1	35	2.4
Αισιοδοξία (Optimism)	5.71%<br>2	2.86%<br>1	0.00%<br>0	0.00%<br>0	5.71%<br>2	8.57%<br>3	11.43%<br>4	31.43%<br>11	34.29%<br>12	35	7.31
Πόνος (Pain)	40.00%<br>14	34.29%<br>12	20.00%<br>7	0.00%<br>0	2.86%<br>1	0.00%<br>0	2.86%<br>1	0.00%<br>0	0.00%<br>0	35	2.03
Σεβασμός (Respect)	2.86%<br>1	2.86%<br>1	0.00%<br>0	0.00%<br>0	0.00%<br>0	0.00%<br>0	28.57%<br>10	31.43%<br>11	34.29%<br>12	35	7.69
Συμπάθεια (Sympathy)	25.71%<br>9	31.43%<br>11	22.86%<br>8	5.71%<br>2	2.86%<br>1	0.00%<br>0	0.00%<br>0	5.71%<br>2	5.71%<br>2	35	2.91
Απελπισία (Desperation)	14.29%<br>5	20.00%<br>7	45.71%<br>16	14.29%<br>5	2.86%<br>1	0.00%<br>0	0.00%<br>0	0.00%<br>0	2.86%<br>1	35	2.89
Απώλεια (Loss)	2.94%<br>1	0.00%<br>0	0.00%<br>0	0.00%<br>0	0.00%<br>0	2.94%<br>1	26.47%<br>9	23.53%<br>8	44.12%<br>15	34	7.91
Πένθος (Mourning)	2.78%<br>1	0.00%<br>0	0.00%<br>0	2.78%<br>1	0.00%<br>0	2.78%<br>1	13.89%<br>5	19.44%<br>7	58.33%<br>21	36	8.08
Φόβος (Fear)	2.86%<br>1	0.00%<br>0	0.00%<br>0	0.00%<br>0	2.86%<br>1	0.00%<br>0	17.14%<br>6	14.29%<br>5	62.86%<br>22	35	8.17
Έρωτας (Love)	2.86%<br>1	5.71%<br>2	0.00%<br>0	0.00%<br>0	2.86%<br>1	5.71%<br>2	34.29%<br>12	28.57%<br>10	20.00%<br>7	35	7.11

Table 9: Valence rating of the words selected

References

[1] D. A. Handweker, J. Ollinger, and M. Esposito, "Variation of BOLD hemodynamic responses across subjects and brain regions and their effects on statistical analyses," *NeuroImage*, vol. 21, no. 4, pp. 1639–1651, Apr. 2004.

[2] K. J. Friston, O. Josephs, G. Rees, and R. Turner, "Nonlinear event-related responses in fMRI," *Magnetic Resonance in Medicine*, vol. 39, no. 1, pp. 41–52, 1998.

[3] A. H. Andersen and W. S. Rayens, "Structure-seeking multilinear methods for the analysis of fMRI data," *NeuroImage*, vol. 22, no. 2, pp. 728–739, Jun. 2004.

[4] C. Chatzichristos, E. Kofidis, M. M. Moreno, and S. Theodoridis, "Blind fMRI source unmixing via higher-order tensor decompositions," *J. Neuroscience Methods*, vol. 315, pp. 17–47, Mar. 2019.

[5] L. De Lathauwer, "Decompositions of a higher-order tensor in block terms–Part II: Definitions and uniqueness," *SIAM J. Matrix Anal. Appl.*, vol. 30, no. 3, pp. 1033–1066, Sep. 2008.

[6] C. Chatzichristos, E. Kofidis, Y. Kopsinis, M. M. Moreno, and S. Theodoridis, "Higher-order block term decomposition for spatially folded fMRI data," in *Proc. of Latent Variable Analysis and Signal Separation Conf. (LVA/ICA)*, Grenoble, France, Feb. 2017.

[7] V. D. Calhoun, T. Adalı, T. Pearlson, and J. Pekar, "A method for making group inferences from functional MRI data using Independent Component Analysis," *Human Brain Mapp.*, vol. 14, no. 3, pp. 140–151, Nov. 2001.

[8] A. J. Bell and T. J. Sejnowski, "An information maximisation approach to blind separation and blind deconvolution," *Neural Comput.*, vol. 7, no. 6, pp. 1129–1159, Nov. 1995.

[9] Medical Image Analysis Lab (MIALAB), "Group ICA of fMRI Toolbox (GIFT)." [Online]. Available: http://mialab.mrn.org/software/gift/index.html

[10] V. D. Calhoun and T. Adalı, "Unmixing fMRI with independent component analysis," *IEEE Eng. Med. Biol. Mag.*, vol. 25, no. 2, pp. 79–90, Mar. 2006.

[11] N. Vervliet, O. Debals, L. Sorber, M. Van Barel, and L. De Lathauwer, "Tensorlab 3.0," Mar. 2016. [Online]. Available: http://www.tensorlab.net
