# Supplementary material for: Emojis influence autobiographical memory retrieval from reading words: An fMRI-based study
Source: PLoS One. 2020 Jul 1;15(7):e0234104. doi: 10.1371/journal.pone.0234104 (PMC7329082; doi:10.1371/journal.pone.0234104)
Supplement: S2 Text — The consent form which was signed from the volunteers in the study. (PDF) [file pone.0234104.s002.pdf]

The medical imaging technique that it will be used, Functional Magnetic Resonance Imaging (fMRI), uses one powerful magnetic field and radiofrequencies in order to acquire detailed and high quality images of human tissues and organs, in our case the brain. The participants in the research will not be exposed to radiation.

The research in which you are requested to take part is directed by the professor of Informatics and Telecommunications Department of Kapodistrian University of Athens Dr. Sergios Theodoridis and Dr. Nikos Andreadis, Vioiatriki SAE. In the research participated several scientists, professors, and phd students under the supervision of the aforementioned researchers.

The ethical committees of Bioiatriki health center and Surrey University (UK) have read and approved the current information sheet and the consent form of the participant.

1.1 Name of the study

Smiley. Study of the neural network associated with the identification of persons and words, using fMRI.

1.2 Purpose of the study

The intention of the researchers is to map the areas of recognition of persons and words, and the memories retrieved from those on healthy human brains

1.3 Can I participate in this study?

You participation in the study will be decided based on the information we will get from an interview. The basic perquisites for the participants are:

1) To be from 18 to 65 years old.
2) They shall not have a physical, neurological or psychiatric disease or any other serious condition that can be judged as factor of exclusion from the study by the researchers
3) They shall not be in pregnancy.
4) They shall have normal or fixed vision (without the use of glasses).
5) They shall not have a pacemaker or stent inserted in their heart or metallic implants and fragments in their head or other part of their body.
6) They shall not have neurological stimulators
7) To sing the consent form that they want to participate in the study

1.4 Is any special preparation needed in order to participate in the study?

Generally no special preparation is needed at Magnetic Resonance Imaging. You can eat and drink normally before your fMRI exam; as well as normally receive any regular medication. You will just be requested to fill in a consent form which includes some safety questions, and to remove any metal objects that you may bear.

1.5 Why shall I be interested in participating in this study?

It is a safe and painless method that gives you the opportunity to participate in scientific efforts to enrich our knowledge about the cognitive factors and brain parameters associated with the development and maintenance of symptoms of emotional disorders such as depression. This knowledge can be used in future for effective diagnosis and treatment of affective disorders.

1.5 I am obliged to participate in this survey? If I decide to participate, can I change my mind and leave before completion?

Participation in this study is voluntary. You can leave the study at any time you want, without giving any explanations.

1.6 What exactly will happen to me, and what procedures I would undergo, if I decide to participate in this study?

The study and the examination will take place in Bioiatriki. You will be asked to come once at the diagnostic center for about 45 minutes. For research purposes you will undergo functional magnetic resonance imaging (fMRI). The test is non-invasive and takes about 30 minutes.

For the examination you will be asked to lie on a soft table and will be asked to be as comfortable as possible. You will be given an alarm button that you can press in case you need to contact the researchers during the examination, or if for any reason you want to stop the examination. During the research process you will hear repeated loud sounds and humming. These noises are normal. You will not feel anything. The machine is a cylinder open at both ends.

1.7 What kind of side effects can I have in this research, and what researchers do to minimize the chances of side effects?

The fMRI is considered a non-invasive method of evaluating brain function. There are no known risks or side effects associated with this test. The magnetic imaging (MRI) uses a combination of powerful magnets and radio waves to acquire pictures of high resolution.

At MRI is not used at all radiation (as in a CT scanner in the x-ray) and there are no known side effects.

1.8 What will the researchers do in order to ensure that the information you get from me (exams, video, etc.) will not be passed to third parties?

All information and research data will remain locked in the files of Department of Informatics and Telecommunications. Only researchers at the department will have your personal information identifying you (name, email address, etc.), while the study results will be published without any evidence which will reveal your identity. None (except from the researchers) will know that you have participated in the study or will have any information that would reveal your identity. Even in possible collaboration with other universities, or research centers, the data will be given without including any personal information.

1.9 What can I personally expect from my participation in this study?

You participate in a study which uses a painless and harmless method of evaluating brain function. This study will give you the opportunity to have a free fMRI scan and keep the results in your medical records.

1.10 Which are the possible benefits from the study for other people?

The benefits are related to the enrichment of our knowledge of the cognitive factors and brain parameters associated with the development and maintenance of symptoms of emotional disorders such as depression. This knowledge can be used in future for effective diagnosis and treatment of affective disorders.

1.11 What will researchers do if I injure myself or have side effects from the study?

You will receive immediate care from the doctor who will be present and the nursing staff of the Medical Imaging Department of Bioiatriki.

1.12 Will I be paid for my participation in the study?

You will be not paid for your participation.

1.13 Is there any charge for me or for my insurance company for this examination or for this study?

No you will not be requested to pay anything during this study.

1.14 During my participating in this study what can I do if I want to learn more about the research or to complain about the way I have been treated

You can communicate with the researchers

Dr Sergios Theodoridis, stheodor@di.uoa.gr ,

Dr Giannis Kopsinis, kopsinis@ieee.org , 2107275104

Dr Nikolaos Andreadis. Neuropsychologis, coordinator of fMRI at the Department of Medical Imaging of Bioatriki and Fellow of Surgical Epilepsy Unit of Neurosurgery Clinic of the Medical School of Athens: 6973432744, andreadis@neurosurgery.org.gr

1.15 If I decide not to participate in this study will happen to me?

There will not be any consequences if you do not participate.

1.16 Who will keep the consent form that I will sign?

You will sign to copies. The one signed copy will be kept at BioIatriki while you will keep the second one.

2. CONSENT FORM

2.1 Identity of the participant

Full name: ____

Date of birth: ___________________________________

Telephone: _________________________________

Email: ________________________________

Serial number: _______________________________________

2.2 Participant's consent form.

Name of the study: Smiley. Study of the neural network associated with the identification of persons and words using fMRI

Place of the examination: ______________________________________

	Please circle
I was informed both written and orally from the researcher for the purpose, process and expected outcomes of the studies and also on possible advantages and disadvantages or side effects from my participation.	**YES/ NO**
I have read the information sheet that has been given to me for the study in which I will participate.	**YES/ NO**
I had adequate time to understand the details and ask questions whenever I considered it necessary in order to give my consent well informed. I can keep the information sheet and get a copy of my written consent.	**YES/ NO**
I will participate in the study as a volunteer, I do not claim any financial reward and my examination with the MRI scanner is not for finding a specific disease.	**YES/ NO**
I am aware that the data of the examination will be used for research and to this end will be saved and edited with computers. There is the possibility of storage and future use in similar research.	**YES/ NO**
I know that I am not obliged to participate in this study and at any time I can leave without explaining my decision to stop.	**YES/ NO**
I know the possibility and agree that the data of my examination may be shared with other universities and research centers. My anonymity will be ensured by the BioIatriki and the National University and any publications of the research findings will not include personal details.	**YES/ NO**
I do not know any reason (medical or psychological) due to which I should not participate in the study.	**YES/ NO**

- I am 18-65 years old ☐
- I do not have in my body a pacemaker ☐
- I do not have in my body any metal stent ☐
- I do not have any cerebral valve of encephalospinal liquid. ☐
- I do not have any hearing device in any of my ears. ☐

- I do not have any metal implant. (tooth implant, spiral etc). ☐
- I never had any accident which have included metal fragments or bullets which have entered my body. ☐
- I do not have body piercing which cannot be removed. ☐
- I do not have a tattoo. ☐
- I do not wear colored eye lenses. ☐
- I do not have any problems with claustrophobia. ☐
- I have not underwent any surgery the last six weeks. ☐
- I do not suffer from epileptic seizures. ☐
- I am not pregnant (or during breast feeding period). ☐

Signature ____________________ Date ____________________

Full name (in capital letters) ____

2.3 Researcher's statement

I gave to Mr/ Ms _______________________________________ the information sheet of the aforementioned study, which in my opinion is accurate and sufficient so that the participant can be fully aware of the nature, risks, and benefits of study and the rights of those involved. I did not put any pressure or improper influence to the participant in order to sign. I certify the signature of the consent form the participante

Signature ___________________________

Date __________________________

Full name (in capitals) ____
