# Supplementary figures and images for: Emojis influence autobiographical memory retrieval from reading words: An fMRI-based study
Source: PLoS One. 2020 Jul 1;15(7):e0234104. doi: 10.1371/journal.pone.0234104 (PMC7329082; doi:10.1371/journal.pone.0234104)

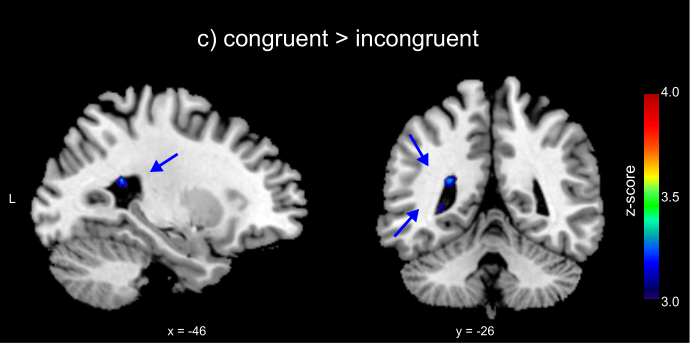

Supplement: S1 Fig — (TIFF) [file pone.0234104.s003.tiff]
