# Supplementary material for: Emojis influence autobiographical memory retrieval from reading words: An fMRI-based study
Source: PLoS One. 2020 Jul 1;15(7):e0234104. doi: 10.1371/journal.pone.0234104 (PMC7329082; doi:10.1371/journal.pone.0234104)
Supplement: Attachment — Submitted filename: Emojis_Review.pdf [file pone.0234104.s005.pdf]

Review Summary

This document contains the answers to the main concerns raised by the Reviewers.

Reviewer #1

General

The use of word “sentimental” is confusing. After reading the article, it becomes clear that it means “emotional.” It is my understanding that, in English, however, “sentimental” could mean more specific feelings (e.g., sadness, or nostalgia) than broadly construed emotion. Thus, I suggest that authors change the word to “emotional” so there is no confusion to readers.

We would like to thank the reviewer for this comment. We have changed the word “sentimental” to the word “emotional” throughout the text.

Abstract

- It should be corrected to indicate that the final sample was 15. As of now, it can mislead readers to think that the final sample was 20.

We have changed the size of the final sample in the Abstract to 15 as suggested by the reviewer.

Introduction

1. In the intro, there is nothing mentioned about autobiographical memory retrieval (in relation to the role of emojis) even though the AMT was the main task. It would be important to make it clear how authors think the task used in the study (AMT with cues with emotional incongruent contents) addresses the aim of the study specifically.

2. To tighten up the intro, you can cut down some of the “emoticon” related content as it is less directly relevant to the present study

We would like to thank the reviewer for his/her comment. We have removed lots of the “emoticon” related content and added more information about the autobiographical memory retrieval.

Methods/Results

1. Please add the ratings scales used for Valence and Arousal.

The ratings scale of Valence and Arousal have been added. The text has been transformed as follows:

“for the valance, arousal, imageability, and familiarity, again using SurveyMonkey's software (using the same Likert scale from 1 to 10)”

2. The AMT task should be more clearly described for readers who are not familiar with the task. In the AMT task, is there any way to validate that participants actually retrieved an autographical memory in response to a given cue when they pressed the button?

Following the previous comment of the reviewer we have added extra information about the AMT task in the introduction. All the participants were interviewed after the fMRI acquisition about the memories retrieved. All of them stated that indeed when they pressed the button they retrieved a memory whereas in the cases that they did not press the button it was because it was not possible for them to retrieve a clear memory. The text in the paper has been correspondingly rephrased in order to make this clear.

When interviewed after the scanning, those subjects who did not press the button on time reported that they did not manage to retrieve a clear memory for those cases, while in the cases that they did press the button they have.}

3. Authors narrowed down the contrasts of interest into the following two: 1) incongruent vs. congruent, 2) word+emoji vs. peudowords. Please provide a clear rationale for looking into these contrasts – i.e., what does each contrast gets at in relation to the aim of the study? Also, for the first contrast, does it mean incongruent > congruent? Please make it clear as it could be misinterpreted that you looked at both ways (incongruent > congruent, congruent > incongruent). The same for the second contrast.

We would like to thank the reviewer for this comment. We have included within the main text an explanation regarding the rationale behind the selected contrasts and their implication in this study. Furthermore, the definition of the contrasts has been changed: congruent > incongruent, incongruent> congruent etc.

4. Please describe “Threshold-Free Cluster Enhancement” approach either in the text or in a footnote.

Since the Threshold-Free Cluster Enhancement method is not so common in the field and since both reviewers have concerns about the method (TFCE can not be used in Blind Methods) we chose to replace the TFCE method with a standard t-test method.

5. For the GLM results, please rewrite the findings to describe saying "xxx regions were more activated in incongruent than congruent trials" if that's what authors meant.

Following the comment of the reviewer the findings description has been rewritten.

6. Figure 4. (a) should be Incongruent vs. Congruent, correct?

We would like to thank the reviewer for this comment. There was a typo there and it has been corrected.

7. In addition to GLM, authors presented results from alternative approaches (i.e., Infomax, BTD). First of all, it is unclear how Infomax approaches different from BTD. Next, I understand the values of using alternative approaches in general. However, given the very straightforward block-design task authors used and the overall similar results driven from the alternative methods, I am not sure how alternative approaches help addressing the research questions in addition to GLM. Much space in the methods/results section is devoted to describing these alternative approaches, but authors used little space in the discussion interpreting the related findings. If authors think that these approaches added something unique to the study different from GLM, then please make it clear. If not, I think the related contents can go to supplementary.

8. Results section for the alternative approaches (i.e., infomax, BTD) needs to be re-written to make it easier for readers to understand/follow what common and unique effects came out of each approach.

9. *It is unclear what authors mean by "Visual" in Infomax and BTD analyses.*.

We would like to thank the reviewer for these comments concerning the blind methods. Following his/her suggestions we have done a major restructuring of the text and the format of the paper with respect to the Blind Source Separation Methods. Since the findings from ICA were not significantly different, they have been moved to the Supplementary Material. In the main paper we have only retained the results of BTD, where the activation regarding the word+emoji task and pseudoword task were of added value and complemented the results of FSL. Furthemore, BTD helped to identify significant activity in some extra areas of interest such as in the cingulate gyrus.

The main difference between ICA and BTD lies in the fact that ICA uses the assumption of independence of the different sources while BTD is a deterministic method based on the more realistic assumption of the low rankness of the spatial maps of activation.

Discussion

1. My biggest concern is that, as of now, the discussion section appears to be just a summary of the results. It is unclear how authors interpret the findings and why they think these are significant in understanding the role of emojis, as a type of communication withthe CMC. It would be critical to connecting the current findings back to the research questions authors raised in the intro.

2. Whereas the introduction focuses on reviewing existing findings on the role of emojis as a communication tool, and its congruence effect, the discussion rather focuses on the other contrast authors examined (word+emoji vs. peudowords). This contrast does not specifically tell us about the unique influence of emojis however. What is the implication of the findings from this contrast?

3. Again, relatively little is devoted to interpreting the findings from the incongruent vs. congruent contrast. It remains unclear how authors interpret the behavioral and neural finding from this contrast.

We understand these concerns of the reviewer. We have seriously restructured the whole discussion section. Thus, we have tried to connect the findings to the initial questions that we raised in the introduction as suggested by the reviewer. Furthermore, we have modified the discussion section so as to provide a clear interpretation regarding the different contrasts and their implications for this study.

4. Also, generally, a more basic contrast should be discussed first than go into a more specific contrast. In this case, I think authors should discuss findings from 1) word+emoji vs. peudowords first and then move on to discussing findings from 2) incongruent vs. congruent.

Please keep the order of discussion of the contrasts (whichever makes most sense in relation to the research questions) throughout the Intro, Methods, Results, and Discussion.

We would like to thank the reviewer for this comment. We have changed the order of the contrasts accordingly.

5. Authors should devote at least one paragraph to discuss about limitations of the study (including small sample size) and the future directions.

We have added such a limitation paragraph in the last part of the discussion section, following the suggestion of the reviewer.

Editor’s Review

In addition to all of the points made by the first reviewer, I would like you to address the following in your revision:

Abstract

"and trigger functional brain networks related to face recognition, contrary to emoticons"

The study doesn't look at emoticons, so this isn't a conclusion that can be reached here.

We would like to thank you for this comment. We have rephrased the sentence to

"contrary to what previous studies have found out for emoticons"

Introduction - *the introduction contains a considerable amount of material that is superfluous to any of the brain or behavior predictions. Some info on the history of emoticons is (perhaps) useful for setting the stage for the rest of the paper, but the first 3 paragraphs include a lot of information about emojis and emoticons that aren't necessary. This comes at the seeming expense of discussing the relatively wide research on emotional congruence on fMRI activation and memory performance, or why such a question would be asked in the first place. Likewise there is a considerably amount of work done to describe differences between fMRI and EEG that are more encyclopedic (spatial vs temporal resolution trade-offs) than they are justifying the study per se. Finally, there is a decent amount of hopping back and forth between EEG and fMRI work on emoticons or emojis, but how exactly it motivates the present work is not always clear. I would like to see the introduction substantially reworked to connect better with the affective science literature on emotional congruence and effects of emotion and emotional congruence on memory. It would also be good to more tightly integrate the previous emoticon and emoji research with the affective science research on emotional congruence when describing the motivations for the present study. Simply put, the study needs to be more clearly integrated with the existing literature and better motivated.*

The introduction has been rewritten in the revised version. We reduced the information regarding emoticons and emojis that was superfluous. We have also included some extra information regarding similar studies in fMRI, such as AMT studies or studies with incongruency effects. Furthermore, we presented more details regarding the objectives and implications of the proposed study.

Methods- *missing some parameters for your EPI acquisition, such as flip angle*

In the revised version, we have defined the EPI acquisition parameters that were missing.

" we defined three different contrasts: incongruent (HS+SH) vs. congruent (HH+SS), congruent vs. incongruent and word+emoji vs. pseudowords (pseudowords have been used as the baseline regressors)"

I've read this a bunch of times and I am having a hard time figuring out what was actually tested. The contrasts should be clearly enumerated. Is it 1) congruent vs incongruent 2) congruent and incongruent vs pseudo words 3) word+emoji vs pseudo words?

We have clarified the definition of the contrasts. We think that instead of using “vs” the “>” symbol is more representative for the contrasts under investigation.

"Although the initial assumption was that the congruent vs. incongruent contrast could have also provided valuable information, Therefore, based on the findings of the first-level analysis and the assumptions made, only two contrasts were taken into consideration at the second (group) level:"

This isn't at all proper procedure in fMRI analysis (testing whether an effect exists at level one before moving to a second level). For one, it is a biased analysis as it uses information about the contrast to select results to report (see Kriegesorte's paper on double dipping). Second, the within subject variance is the only variance of interest in the first-level model, but plays a minor role in the second level model where the between subject variance is the primary variance of interest. Thus the only test that makes any sense to look at is the second level analysis. If a contrast is of interest at any level, it needs to be examined and reported at the second level.

We agree with this comment. We have also analyzed the congruent vs incongruent contrast in the multi-subject level , but it was obviously not reported clearly enough. In the revised version of the paper, we have reported the results regarding this contrast properly. Moreover, we have added some extra details about their implications for the analysis.

There is a lot of work being done in the methods to justify the blind source separation. To the degree that this is novel and a major part of the present work, it needs to be covered prior to the methods, in the intro. In the methods, the implementation and software, etc. should be described but a long description of the method and justification of it relative to the GLM is less appropriate. From my perspective, there isn't much new happening here as tensor ICA is implemented in many commonly available fMRI analysis packages. Perhaps the BTD algorithm is less frequently used. In either case, this section could be streamlined to convey what was done as opposed to having extensive explanation of how the method works and justification for it. To the degree those are necessary, they should be moved to a more appropriate section.

We would like to thank the editor for these comments concerning the blind methods. Following his/her suggestions we have done a major restructuring of the text and the format of the paper with respect to the Blind Source Separation Methods. Since the findings from ICA were not significantly different, they have been moved to the Supplementary Material. In the main paper we have only retained the results of BTD, where the activation regarding the word+emoji task and pseudoword task were of added value and complemented the results of FSL. Furthemore, BTD helped to identify significant activity in some extra areas of interest such as in the cingulate gyrus.

The main difference between ICA and BTD lies in the fact that ICA uses the assumption of independence of the different sources while BTD is a deterministic method based on the more realistic assumption of the low rankness of the spatial maps of activation.

-Results The ANOVA, the t-test, and Tukey test are somewhat redundant. It's conventional to report an omnibus ANOVA prior to a post hoc test for a difference between means (Tukey or t-test). Here it seems to make the most sense to simply report the Tukey test after the ANOVA as it is clearer about what is driving the observed ANOVA and difference between congruent and incongruent than the first reported t-test.

Thank you for this comment. The first t-test has been removed.

The fMRI analysis starts by saying that it is reporting tfce corrected results at a threshold of p < .05 but then the figure reports z > 3, which is reported as p < .01, corrected. I don't know how to reconcile those two things but z > 3 is closer to a one-tailed uncorrected p of p < .001 (~3.09). If standard tfce p < .05 is used, then that should be what is depicted in the figure. Likewise, the table reports significant clusters with k > 20, but that again, isn't really how tfce works, which gives voxel-wise corrected p-values. Any cluster that meets the cut-off chosen in the study should be reported. For the GICA and BTD results, it's not clear why TFCE wasn't used and the z > 3 (p < .01) issue arises again. Typically one would threshold all whole brain maps in a study using approximately the same criteria. Z > 3 and 20 voxels alone isn't a valid correction for multiple comparisons; something else needs to be used.

First, the p<0.05 mentioned initially was a typo. We meant that we used a FWE correction at p<0.05 and then all the maps were thresholded with z>3 (p<.001). Second, the clusters with k>20 were reported in the table to save space. In the figures all the clusters were depicted.

In the revised version, we decided to redo everything and clarify everything better: First, we implemented a standard t-test to assess for significant activity for the different contrast, instead of TFCE , since the t-test is more commonly used in GLM-based studies. Second, the obtained significant activation maps were thresholded for significance at z>3.09, and they were corrected for Family-Wise Error at p<0.05. Finally, all the clusters have been reported in the tables (as well as in the figures), to provide a clear picture of all the obtained results.

For BTD, the significant activation maps were also thresholded for significance at z>3.09. BTD is a deterministic method but of course when computing the z maps the standard deviation of the noise per voxel is taken into consideration. Hence no FWE error correction is needed [48,57].

I'm confused again about what was actually tested with respect to Fig 4 as the methods state that the congruent vs non-congruent contrast was abandoned due to not finding first level results. However, Fig 4 states that panel a is congruent vs non-congruent.

We would like to apologize for this mistake. Indeed, that figure contained a typo in the caption; it should be incongruent > congruent. We have fixed this typo in the revised version.

Discussion-

Need clearer limitations - 15 subjects is the total sample size and is small given current standards in fMRI research. Using the same sequence for all or most subjects, particularly one with a slow block design like the current is risky because it makes consistent low frequency information like scanner noise correlate with the signal. Thus although using the same sequences is necessary for tensor decomposition, it needs to also be listed as a possible limitation.

We have added such a paragraph, at the end of the discussion section, following your suggestion. We have tried to decrease the correlation with the low frequency signal since we used random timings between the different blocks and also the appearance of the different conditions per subject is random (but the same among subjects). We can of course understand the concern of the editor and this has been added in the paper as a possible limitation (along with that of the number of subjects).

It's also not clear how the contrasts tested and the design of the task led themselves to studying emojis per se as emoticons or other pictorial emotional stimuli are not used. Emojis are only compared to words and pseudo words, which means that many of the differences may reflect simple differences in language-based vs pictorial representations and congruent vs incongruent emotional stimuli as opposed to anything specific to the representation of emojis. "emojis manage to trigger also particular face-emotion recognition areas."
As per the abstract comment, this can't be stated with any certainty here as it revolves around what Poldrack refers to as a "reverse inference". The fusiform represents a lot of different things, and without an independent localizer showing that this is the fusiform face area, it's impossible to say with any certainty that the emojis are triggering a face specific region.

We can understand the skepticism of the editor. An independent localizer could have indeed been used, but we have already included the pseudowords and the fixation cross to disengage the subjects from the previous memory (we consider it as a more important possible drawback or bias in the results) and hence the length of the experiment had been already quite long (approximately 18 min). Adding an independent localizer (e.g., identical stimuli without emojis) would further increase the duration of the experiment to around 40 min. Consequently, the probability of subject dropouts would increase. Since we had limited sources for multiple fMRI tests, we opted to include the pseudowords but not the independent localizer. What has played a critical role in our decision is that, in the papers of Yuasu et al. with similar task (the main difference being emoticons vs. emojis), no activation in the fusiform gyrus was documented. However, we have found evidence that potential areas regarding face recognition are involved, such as the inferior fusiform gyrus (BA37) or

the amygdala. Furthermore, other areas such as the temporal pole plays a role in emotion recognition from emotional faces and images. Consequently, in order to address this issue, we have included a subsection ("Face-recognition" subsection, page 25) within the discussion where we detail the implications and limitations of the analysis of this study regarding face recognition.

Despite all of the effort to explain and justify use of the ICA approaches, it's not clear what conclusions one should reach from them in the discussion or why they were done (other than to do something different from standard fMRI analysis). As per above, the theoretical merit (if any of these analyses) needs to be clearer and connect to the conclusions/discussion (as well as the intro). With the small discussion as is, I don't really understand what these methods add in this case as they aren't substantially different for any of the contrasts where emojis matter.
The discussion should again touch on how these results relate to emotional congruence literature more broadly.

We understand these concerns of the editor. We have seriously restructured the whole discussion section. Thus, we have tried to connect the findings to the initial questions that we raised in the introduction as suggested. Furthermore, we have modified the discussion section so as to provide a clear interpretation regarding the different contrasts and their implications for this study.

Minor grammatical errors

Thank you for pointing out these errors. They all have been fixed.
