# Supplementary material for: Emojis influence autobiographical memory retrieval from reading words: An fMRI-based study
Source: PLoS One. 2020 Jul 1;15(7):e0234104. doi: 10.1371/journal.pone.0234104 (PMC7329082; doi:10.1371/journal.pone.0234104)
Supplement: Attachment — Submitted filename: Emojis_MinorReview.pdf [file pone.0234104.s006.pdf]

Response to the reviewers

We would like to thank the editor and the anoynymous reviwer for their constructive comments. We have made an effort to to respond to all of the min a satisfactory manner

---

There are a number of minor clarification and language issues that should be corrected before acceptance of the manuscript. I'd encourage employing the services of an English language expert or copy editor as well before re-submitting as there are a number of sentences throughout the manuscript that are difficult to parse and could benefit from some fine tuning.

Comment #1

"The description of stroop tasks in the introduction is particularly unclear, for example"

We have accordingly modified the abstract and the conclusions section. Specifically the stroop task description has been changed as follows:

"For example, several works have examined the response of the brain to semantic conflicts such as the Stroop word-color effect [22-24]. Put succinctly, the classical Stroop word-color effect relies on the observation that naming the ink color of a word takes longer and is more prone to errors if the ink does not match the color of the word [25] (e.g., naming the ink color of the word blue takes more time if the ink of the word is not blue). In those studies, the analysis of the fMRI data showed that, compared to congruent word-color stimuli, incongruent combinations activated the paracingulate gyrus, the middle and inferior frontal gyrus (including BA47), the lateral occipital the precuneus and the anterior cingulate. Among these areas, the anterior cingulate gyrus plays a crucial role in ambiguity resolution, as [26, 27] also reported."

Comment #2

"'and trigger several functional brain networks related to word processing"

Trigger isn't an appropriate description here. Perhaps just say that emojis lead to activation in the specific brain regions that are found to be activated in the study. As discussed in my previous review, in fMRI reporting we like to keep the amount of "reverse" inferences" to a minimum, perhaps outside of discussion. Here many readers would have questions about what exact brain regions are activated for word processing, emotion cognition, and memory retrieval as many regions do these things. As discussed before, it also isn't possible to say that these regions were doing these things in this context because none of the contrasts really specifically isolate these functions. Thus, particularly in the abstract, the actual results need to be spelled out. Not only should the specific brain regions be written here, but the specific test should be listed before describing the conclusions, like "Compared to pseudo words, words and emojis activated regions x, y, z" "There was more activation in regions x, y, z for congruent compared to incongruent emoji and word combinations" These will help readers understand what was actually found and make correct inferences about the results.

We would like to thank the editor for this comment. We have modified the abstract accordingly:

"The analysis of the fMRI data showed that emotional incongruity among word+emoji combinations activated the Broca's area (BA44 and BA45) in both hemispheres, the Supplementary Motor Area (SMA) and the inferior prefrontal cortex (BA47), compared to congruent combinations. Furthermore, compared to pseudowords, word+emoji combinations activated the left Broca's area (BA44 and BA45), the amygdala, the right temporal pole (BA48) and several frontal regions including the SMA and the inferior prefrontal cortex."

Comment #3

"delays in the memory retrieval"

No "the" is needed. Just delays in memory retrieval. However, it would be more accurate to say that it led to longer reaction times. Like above, the conclusion that this is memory retrieval delays per se is not a sound inference.

We have changed the phrase accordingly.

"led to longer reaction times in memory retrieval"

Comment #4

The authors should replace the words neurological or neurology with neurophysiological or neurobiology in the majority of places in the manuscript. neurology is a specific medical discipline, which focuses brain pathology, and thus saying "neurological" gives the impression that patient work might be presented, which it is not. The current manuscript is more in line with neurophysiology, neuroscience, neurobiology, or neuroimaging (any of which would be more appropriate to list than neurological)

neurological findings -> neurophysiological data

neurological -> neurophysiological (in many places throughout the manuscript)

We have changed the words as suggested by the editor

Comment #5

"Several EEG studies have pointed out the emotional content of emoticons and emojis"

It seems like emotional content of emoticons and emojis is somewhat of a given that doesn't need imaging to establish. Is the point of this sentence that" EEG studies have identified neurobiological processes associated with processing emotion in emoticons and emojis"?

We modified the text as follows:

"Several EEG studies have pointed out that emoticons and emojis bear an emotional content, similarly to words"

Comment #05

"whether the emoticons are processed"

whether emoticons are processed

The word “the” has been removed as suggested.

Comment #6

"iii) since no significant activity
was detected at the fusiform gyrus or the posterior cingulate gyrus,emoticons do not carry clear semantic content, in contrast to nouns or adjectives."

This isn't really a valid inference as fusiform and posterior cingulate gyrus aren't necessarily the only semantic processing regions (or even the most likely semantic processing regions) and lack of activation differences are like any other statistical test; it doesn't mean you can accept the null when a brain region doesn't activate

This has been pointed out by the authors of the paper Yuasa et al. It is what the authors have stated. We have tried to clarify this in the text by adding the following phrases:

“as it is stated by the authors” and *“ they assume”*

Comment #7

"The initial assumption of our study is that since the emoticons influence the reading of complete sentences, they will also influence the memory retrieval process since it is documented that they have also emotional content."

Clause structure of this sentence is complex - there are two "since"s in it. Should be simplified or split into two.

We have adapted the aforementioned sentence, following the recommendation of the editor as:
“The initial assumption of our study is that since the emoticons influence the reading of complete sentences (due to their emotional content), they will also influence the memory retrieval process.”

Comment #8

In the fmri acquisition section, there appears to be a paragraph more about the fmri task procedure. That should go in the section with the task.

We would like to thank the editor for this comment, indeed this paragraph was misplaced.

Comment #9

There is still inconsistency in the z's listed as significant, even within the FEAT analysis section. For example, one says $z > 3.09$ the other says $z > 3.01$, the former is closer to the

standard $p < .001$ one-tailed cutoff for the z-distribution. The authors should use a single z cutoff.

A single cutoff has been used, namely $z>3.09$. We just missed to correct the specific value at one point in the text. Thank you for pointig this out.

Comments #10 and #12

We would like to thank the editor for his careful reading. We have corrected all the grammar and language mistakes pointed out.

Comment #11

"$z > 2.32$"

Why was z of 2.32 chosen? This doesn't correspond to any of the defaults in FSL where a z of 2.3 is conventionally used as the default threshold

$Z>2.32$ ($p=0.01$ uncorrected) has been used because it is a commonly used, relaxed threshold. Indeed $Z>2.3$ is the default value of fsl but it does not accurately correspond to $p=0.01$.
