# Supplementary material for: Emojis influence autobiographical memory retrieval from reading words: An fMRI-based study
Source: PLoS One. 2020 Jul 1;15(7):e0234104. doi: 10.1371/journal.pone.0234104 (PMC7329082; doi:10.1371/journal.pone.0234104)
Supplement: Attachment — Submitted filename: PeerReviewTemplate_JH_plosOne.docx [file pone.0234104.s004.docx]

The focus of this paper is timely, important, and likely to be of keen interest to a broad spectrum of psychology fields. In the section that follows, I provide a few suggestions for further strengthening the manuscript.

General

- The use of word “sentimental” is confusing. After reading the article, it becomes clear that it means “emotional.” It is my understanding that, in English, however, “sentimental” could mean more specific feelings (e.g., sadness, or nostalgia) than broadly construed emotion. Thus, I suggest that authors change the word to “emotional” so there is no confusion to readers.

Abstract

- It should be corrected to indicate that the final sample was 15. As of now, it can mislead readers to think that the final sample was 20.

Introduction

1. In the intro, there is nothing mentioned about autographical memory retrieval (in relation to the role of emojis) even though the AMT was the main task. It would be important to make it clear how authors think the task used in the study (AMT with cues with emotional incongruent contents) addresses the aim of the study specifically.
2. To tighten up the intro, you can cut down some of the “emoticon” related content as it is less directly relevant to the present study.

Methods/Results

1. Please add the ratings scales used for Valence and Arousal.
2. The AMT task should be more clearly described for readers who are not familiar with the task. In the AMT task, is there any way to validate that participants actually retrieved an autographical memory in response to a given cue when they pressed the button?
3. Authors narrowed down the contrasts of interest into the following two: 1) incongruent vs. congruent, 2) word+emoji vs. peudowords. Please provide a clear rationale for looking into these contrasts – i.e., what does each contrast gets at in relation to the aim of the study? Also, for the first contrast, does it mean incongruent > congruent? Please make it clear as it could be misinterpreted that you looked at both ways (incongruent > congruent, congruent > incongruent). The same for the second contrast.
4. Please describe “Threshold-Free Cluster Enhancement” approach either in the text or in a footnote.
5. For the GLM results, please rewrite the findings to describe saying “xxx regions were more activated in incongruent than congruent trials” if that’s what authors meant.
6. Figure 4. (a) should be Incongruent vs. Congruent, correct?
7. In addition to GLM, authors presented results from alternative approaches (i.e., Infomax, BTD). First of all, it is unclear how Infomax approaches different from BTD. Next, I understand the values of using alternative approaches in general. However, given the very straightforward block-design task authors used and the overall similar results driven from the alternative methods, I am not sure how alternative approaches help addressing the research questions in addition to GLM. Much space in the methods/results section is devoted to describing these alternative approaches, but authors used little space in the discussion interpreting the related findings. If authors think that these approaches added something unique to the study different from GLM, then please make it clear. If not, I think the related contents can go to supplementary.
8. Results section for the alternative approaches (i.e., infomax, BTD) needs to be re-written to make it easier for readers to understand/follow what common and unique effects came out of each approach.
9. In is unclear what authors mean by “Visual” in Infomax and BTD analyses.

Discussion

1. My biggest concern is that, as of now, the discussion section appears to be just a summary of the results. It is unclear how authors interpret the findings and why they think these are significant in understanding the role of emojis, as a type of communication within the CMC. It would be critical to connecting the current findings back to the research questions authors raised in the intro.
2. Whereas the introduction focuses on reviewing existing findings on the role of emojis as a communication tool, and its congruence effect, the discussion rather focuses on the other contrast authors examined (word+emoji vs. peudowords). This contrast does not specifically tell us about the unique influence of emotjis however. What is the implication of the findings from this contrast?
3. Again, relatively little is devoted to interpreting the findings from the incongruent vs. congruent contrast. It remains unclear how authors interpret the behavioral and neural finding from this contrast.
4. Also, generally, a more basic contrast should be discussed first than go into a more specific contrast. In this case, I think authors should discuss findings from 1) word+emoji vs. peudowords first and then move on to discussing findings from 2) incongruent vs. congruent. Please keep the order of discussion of the contrasts (whichever makes most sense in relation to the research questions) throughout the Intro, Methods, Results, and Discussion.
5. Authors should devote at least one paragraph to discuss about limitations of the study (including small sample size) and the future directions.
